# Evolution of β-blockers: from anti-anginal drugs to ligand-directed signalling

**DOI:** 10.1016/j.tips.2011.02.010

**Published:** 2011-04

**Authors:** Jillian G. Baker, Stephen J. Hill, Roger J. Summers

**Affiliations:** 1 Institute of Cell Signalling, School of Biomedical Sciences, Medical School, Queen's Medical Centre, Nottingham, UK; 2 Drug Discovery Biology, Monash Institute of Pharmaceutical Sciences, 399, Royal Parade, Parkville, Vic 3052, Australia

## Abstract

Sir James Black developed β-blockers, one of the most useful groups of drugs in use today. Not only are they being used for their original purpose to treat angina and cardiac arrhythmias, but they are also effective therapeutics for hypertension, cardiac failure, glaucoma, migraine and anxiety. Recent studies suggest that they might also prove useful in diseases as diverse as osteoporosis, cancer and malaria. They have also provided some of the most useful tools for pharmacological research that have underpinned the development of concepts such as receptor subtype selectivity, agonism and inverse agonism, and ligand-directed signalling bias. This article examines how β-blockers have evolved and indicates how they might be used in the future.

## Introduction

Sir James Black made a massive contribution to pharmacology by demonstrating that new major classes of drugs could be developed by applying basic knowledge of receptor-driven cell-signalling systems to clinical problems. He developed two new classes of drugs at a time when few specific medical treatments were available: β-blockers* for cardiovascular disease and H2-antihistamines as antacids for gastric and duodenal ulceration [1]. His less well-publicised contribution was to analytical pharmacology, in which many of his pioneering approaches will continue to resonate for many years to come [2]. His first discovery, β-blockers, was stimulated by the sudden death of his father from a myocardial infarction when Sir James was at medical school. He wanted to ‘stop the effects of adrenaline on the heart’ and therefore improve the chest pain of ischaemic heart disease caused by a lack of oxygen in the heart. Propranolol, which he developed while at ICI pharmaceuticals and still in widespread clinical use today, went on to change medical practice worldwide [3]. In this review we examine how β-blockers have evolved from their origin as treatments for angina and cardiac arrhythmias to be effective therapeutics for hypertension, cardiac failure, glaucoma, migraine and anxiety, and discuss the potential for their future development for the treatment of a variety of conditions.

## Early development and clinical uses of β-blockers

In angina, the coronary arteries are partially blocked (usually by atherosclerosis), which reduces blood flow to the heart muscle. Exercise, stress and emotion increase sympathetic drive (which increases the rate and force of contraction), thus requiring increased coronary artery blood flow. If this is not achieved, myocardial ischaemia occurs, resulting in crushing central chest pain typical of angina pectoris. Sir James surmised that blocking the effects of catecholamines would prevent angina by decreasing the workload. Although Ahlquist had proposed in 1948 that widespread adrenaline effects were mediated through two groups of adrenoceptors, α and β [4], the idea of developing a selective β-adrenoceptor antagonist was still quite radical. After joining ICI Pharmaceuticals in 1958, Sir James used the β-adrenoceptor-selective agonist isoprenaline as a template, but became intrigued that a derivative, dichloroisoprenaline, lowered heart rate [5]. The application of some clever medicinal chemistry by John Stephenson resulted in practolol and pronethanol, the first β-blockers (later withdrawn because of oculomucucutaneous syndrome/sclerosing serositis and carcinogenicity). A safer, more potent derivative soon followed, propranolol, which is still considered the prototypical β-adrenoceptor antagonist (Table 1) [6].

The rationale that Sir James used for the development of β-blockers was that by reducing catecholamine-induced effects of myocardial β-adrenoceptor activation, the pain due to angina would be improved. β-Adrenoceptor blockade leads to an increase in work capacity before pain or ischaemia occurs by reducing oxygen consumption by the heart [7]. Another early clinical observation was that pronethalol and propranolol produced hypotension [8,9]. It was also shown that propranolol has anti-arrhythmic properties. Subsequent work established that the anti-hypertensive and anti-arrhythmic properties, together with the reduction in heart rate and cardiac output, are important class actions of β-adrenoceptor antagonists (Table 1) [10].

## Current cardiovascular uses of β-blockers

The number of β-blockers available rapidly increased and became the major first-line therapy for hypertension (atenolol is historically one of the most frequently prescribed of all medicines). Improvements in the symptomatic management of angina were followed by improvements in mortality in acute myocardial infarction (MI) [11] and long-term when given post-MI [12–16]. β-Blockers also reduce arrhythmias after both cardiac and non-cardiac surgery [17–21]. Thus, β-blockers now have important role in improving both mortality and symptom control in ischaemic heart disease, arrhythmias and hypertension (Table 1) [22].

Recent studies suggest that the reduction in hypertension following β-blockade has not resulted in as great a reduction in stroke as other (newer) treatments, but other evidence contests this and β-blockers remain an important treatment for hypertension [16,23–25].

## Heart failure and β-blockers: paradoxical pharmacology?

Initially β-blockers were contraindicated in heart failure. In 1978, Prichard observed: ‘Two important untoward effects from β-adrenergic receptor blocking drugs that should be avoided with foresight in patient selection: heart failure and asthma. Patients in heart failure, or patients with incipient left ventricular insufficiency … are critically dependent on sympathetic activity to the heart to maintain their cardiac output.’ [10]. Indeed, heart failure occurs when the cardiac output is not sufficient to meet the demands of the body so reducing output further with a β-blocker would appear illogical.

However, the healthy human heart also contains functionally well-coupled β_2_-adrenoceptors [26], with 80% of cardiac β-adrenoceptors of the β_1_ and 20% of the β_2_-subtype [27,28]. In heart failure, the number of β_1_-adrenoceptors decreases such that only 60% are β_1_ (and 40% β_2_) [27]. The role of β_3_-adrenoceptors in human myocardium is still not clear [29]. There is also evidence that long-term activation of β_1_-adrenoceptors in animals has more deleterious effects than activation of β_2_-adrenoceptors. Transgenic mice with modest overexpression of β_1_-adrenoceptors rapidly develop cardiac failure and die, whereas β_2_-adrenoceptor overexpression is better tolerated [30]. In humans, use of β_1_-adrenoceptor-selective agonists was associated with an increase in mortality [31] and they are now restricted to short-term maintenance of cardiac output in intensive and coronary care units.

The initial suggestion that β-blockers were beneficial in heart failure was treated with scepticism [32,33], but subsequent large-scale clinical trials confirmed that they prolong longevity. Bisoprolol [34], metoprolol [35], carvedilol [36] and nebivolol [37] (Table 1) are all effective in reducing mortality in patients (see also Kubon *et al*., this issue). As Prichard noted, heart failure is a state with high catecholamine levels. This is useful in the short term because it increases cardiac drive, but is detrimental in the long term and results in myocardial apoptosis, fibrosis and remodelling. The pharmacological basis for the beneficial effects of β-blockers in heart failure seems to be this reduction in catecholamine-driven detrimental changes [22,38,39]. Furthermore, two β-blockers tested in heart failure patients that were not beneficial, bucindolol [40] and xamoterol [41], both have significant intrinsic sympathomimetic activity (ISA) [39,42,43].

## Other clinical uses of β-blockers

β-Blockers (e.g. timolol and betaxolol) are also widely used in glaucoma (raised intraocular pressure) (Table 1) [44]. Propranolol is used for prophylactic treatment of migraine and is as effective as many other treatments available [45]. Interestingly, ISA also seems to make β-blockers less effective in the management of migraine [46]. β-Blockers are also used as anxiolytics in both acute and generalised anxiety disorders, for which their likely mode of action is blockage of the peripheral effects of high circulating levels of catecholamines [47]. Short-acting drugs such as propranolol are also popular among performers to reduce catecholamine-induced tremor during performances. β-Blockers are also used in portal hypertension [48] and benign essential tremor [49] and propranolol is used for symptomatic management in hyperthyroidism (Table 1) [50].

## Future potential clinical uses of β-adrenoceptor antagonists

Osteoporosis is characterised by a decrease in bone mass due to an imbalance between osteoclast bone reabsorption and osteoblast bone formation. This results in bones that are more fragile and easily fractured. Several cross-sectional clinical studies revealed an association between β-adrenoceptor antagonist treatment and reduced risk of bone fracture [51–57]. Some longitudinal studies also suggest that β-adrenoceptor antagonist treatment can have a protective effect [53,58–60]. In rats [61] and mice [62], propranolol treatment increases bone mass, and β-adrenoceptor agonists such as isoprenaline, clenbuterol and salbutamol have the opposite effect [63,64]. The sympathetic nervous system (SNS) is important in the control of bone formation. In conditions associated with reduced SNS activity (e.g. leptin-deficient *ob/ob* mice or dopamine β-hydroxylase-deficient mice) there is high bone density [62]. Removal of the adrenal medulla does not affect bone mass, which suggests that the effect is neuronally regulated. The effects of β-adrenoceptor ligands on bone density are probably mediated by actions on β_2_-adrenoceptors present on osteoblasts [62,65]. However, the role of β-adrenoceptors in bone remodelling is complex and further work is required to reach consensus on the utility of β-adrenoceptor agonists and antagonists for the treatment of osteoporosis [66].

β-Adrenoceptors might also be important in cancer metastasis, because metastatic spread can be inhibited by β-adrenoceptor antagonists. Epidemiological studies have revealed a link between the use of β-adrenoceptor antagonists and reduced cancer risk [67,68]. In a mouse model of breast cancer, activation of the SNS by stress causes a 30-fold increase in metastasis mimicked by subcutaneous administration of isoprenaline and blocked by propranolol [69]. In a model of human ovarian cancer, detachment of cells from the extracellular matrix or from neighbouring cells is associated with apoptosis, a process known as anoikis. Treatment of human ovarian cancer cells with catecholamines reduces anoikis, probably by activating protective focal adhesion kinases (FAKs). Noradrenaline treatment causes increased pFAK^Y397^ phosphorylation and decreased anoikis, which can be blocked by cell pretreatment with FAK siRNA [70]. Similar blocking effects were produced with propranolol or butoxamine or by pretreating cells with β_2_-adrenoceptor siRNA but not with atenolol [70]. The Y397 phosphorylation site on FAK is a high-affinity binding site for the SH2 domain of Src, and phosphorylation of this site following cell exposure to noradrenaline is prevented by the Src inhibitor PP2, but not by the inactive congener PP3 [70]. It will be interesting to determine whether the coupling of β_2_-adrenoceptors to Src involves β-arrestin and internalisation, because it is now possible to identify ligands with a ligand-directed signalling bias† for the β-arrestin and G-protein-coupled pathways (see below) [71].

β-Adrenoceptors also seem to play a role in regulating infection. Merozoite invasion of erythrocytes by the human malaria parasite *Plasmodium falciparum* is enhanced by treatment with β-adrenoceptor agonists and blocked by the antagonist propranolol and inverse agonist ICI118551 (which also reduces the baseline response). The inactive (+)-isomer of propranolol was ineffective against both *in vitro* and *in vivo* infection [72]. In addition to invasion, erythrocyte G_s_ signalling is also required for growth and proliferation of malaria parasites [73]. Interestingly, propranolol was more potent in inhibiting growth than in preventing invasion. Used in combination with existing anti-malarial compounds, propranolol reduced the inhibitory concentrations by five- to ten-fold [73]. In studies examining the inhibitory effects of several β-adrenoceptor antagonists (10 μM) on maturation of *P. falciparum* in *in vitro* cultures, propranolol, alprenolol and ICI118551 were very effective in reducing viability, whereas other antagonists (e.g. nadolol, butoxamine, acebutalol, atenolol and metoprolol) were less effective [73]. The actions of β-adrenoceptor antagonists on various aspects of the life cycle of *P. falciparum* suggest that there might be potential to develop combination therapies with existing anti-malarial drugs.

## β-Blockers and airway disease

There are major concerns about the use of β-blockers in patients with respiratory disease, particularly asthma. Blockade of β_2_-adrenoceptors in the airways (by β-blockers including atenolol, metoprolol, acebutolol, bevantolol, xamoterol, bisoprolol and betaxolol [74–83]) causes bronchoconstriction and reduces the effectiveness of the main rescue treatment for asthma. Even though some blockers are more β_1_-adrenoceptor-selective, this selectivity is poor and escalation of the β-adrenoceptor agonist dose is required to restore lung function. Most studies show a significant decrease in basal lung function in approximately half of patients, with half of patients tolerating a single-dose β-blocker [84]. Similar results were found in studies with concomitant illnesses (e.g. carvedilol in patients with heart failure and asthma) [85]. Several studies reported a rapid decrease in lung function or severe breathlessness following a single dose of β-blocker in some individuals [79,84–86]. Thus, although long-term studies of the effects of β-blockers in asthma patients are lacking, β-blockers are currently absolutely contraindicated in patients with asthma, even for the most β_1_-selective antagonists currently available and despite their life-prolonging effects in cardiovascular disease [87]. Studies of long-term administration of low-dose β-blockers (see also Page, this issue) are in progress [88] to determine if the paradoxical pharmacology observed in congestive heart failure is also evident in asthma [89].

## Pharmacological basis for the clinical actions of β-adrenoceptor antagonists

Many β-blockers now exist and these differ in physicochemical, pharmacokinetic and pharmacodynamic properties. Current drugs vary in their selectivity for β_1_-, β_2_- and β_3_-adrenoceptors, and some, such as carvedilol and labetalol, are also α_1_-adrenoceptor antagonists. Some have partial agonist activity (ISA), local anaesthetic properties (membrane-stabilising activity), K^+^ channel blocking activity or anti-oxidant properties.

## Properties of β-adrenoceptor antagonists

### Subtype selectivity

One of the earliest developments of β-adrenoceptor antagonist drugs was subtype selectivity. The prototypical β-blocker propranolol has similar affinity for β_1_- and β_2_- adrenoceptors and lower affinity for β_3_-adrenoceptors [90,91]. However, even the ‘cardioselective’ β-blockers, a nomenclature based on their selectivity for β_1_-adrenoceptors, are not, because none in clinical use are that selective (13-fold at most) [90,92–94]. Given this fact, there might well be a good case for developing a highly selective β_1_-adrenoceptor antagonist. Such a compound would be potentially useful in patients with asthma and other respiratory disorders who require inhaled β_2_-adrenoceptor agonists to produce life-saving bronchodilatation. Of the six drugs that have been investigated in substantial heart failure trials to date (bisoprolol and nebivolol being the most β_1_-selective, carvedilol having slight β_2_-selectivity), it is not possible to predict what level of selectivity, if at all, is required for maximum beneficial outcomes in heart failure.

Most currently available β-blockers (including propranolol) have low affinity for the β_3_-adrenoceptor. There are, however, a subset of drugs comprising oxprenolol, carazolol, pindolol, nadolol, tertatolol, carteolol, arotinolol and nebivolol that have agonist effects at the β_3_-adrenoceptor that could be responsible for the nitric oxide (NO)-mediated vasodilator properties observed with nebivolol [92,95]. Even SR59230A, claimed to be selective for β_3_-adrenoceptors, has a similar potency at all 3 subtypes [95,96]. Other more recent human selective β_3_-adrenoceptor ligands (such as L748337) do display greater subtype selectivity [95].

### Intrinsic sympathomimetic activity and partial agonism

Some β-blockers are traditionally described as having ISA. These drugs block the stimulatory effects of high-efficacy agonists, such as catecholamines, but stimulate agonist responses of their own. This is evident at both the cellular [95,97–101] and tissue level [42,43,99,102] with acebutolol, carteolol, penbutolol, pindolol, bucindolol and xamoterol, for which it is claimed that bradycardia and bronchoconstriction are less than for other β-adrenoceptor antagonists. However, drugs with ISA are less advantageous in the management of heart failure and migraine.

### Low-affinity state of the β_1_-adrenoceptor

Some β-adrenoceptor antagonists stimulate β_1_-adrenoceptor function by interacting with a low-affinity state of the β_1_-adrenoceptor [103–106] and the β_3_-adrenoceptor [100]. Following initial observations [107,108], most detailed observations have been for CGP12177A [109], but β-adrenoceptor antagonists with similar properties include oxprenolol, alprenolol, carazolol, pindolol and carvedilol. These ligands either stimulate agonist responses at concentrations much higher than those required to fully occupy and block the conventional catecholamine β_1_-adrenoceptor site, or have biphasic concentration–response curves [92,95,100,105,110,111]. Activation of this low-affinity state of the β_1_-adrenoceptor has been demonstrated in model cell systems [99,101,112], cardiomyocytes [113,114], tissues [115], whole animals [116,117] and humans [118]. However, there is currently no therapeutic use for this property of β-adrenoceptor antagonists.

### Inverse agonism

Many β-adrenoceptor antagonists, at least at the β_2_-adrenoceptor, are in fact inverse agonists [119–121] (i.e. rather than just occupying the binding site and thus blocking the actions of agonists, they are associated with conformations of the receptor that turn off signalling [122]).

### Other properties of β-blockers

Local anaesthetic or membrane-stabilising activity is shown by some β-adrenoceptor antagonists, notably propranolol and acebutolol and to a lesser extent pindolol and labetalol. Although this is observed in model systems, it is unlikely to be important in the therapeutic effects of β-blockers because it occurs at much higher concentrations than those normally encountered clinically.

Individual (rather than class effect) properties of certain β-blockers include lipophilicity, K^+^ channel blockade and anti-oxidant properties. Propranolol, timolol and metoprolol are somewhat lipophilic. Sotalol can block K^+^ channels independently of its β-blocking properties. Carvedilol blocks α_1_- and β-adrenoceptors, inhibits apoptosis and possesses antioxidant and free-radical-scavenging actions. Nebivolol causes NO-dependent vasodilation. These properties might contribute to their efficacy in cardiac failure.

## Emerging properties of β-adrenoceptor antagonists: ligand-directed signalling bias

**Figure 1:**
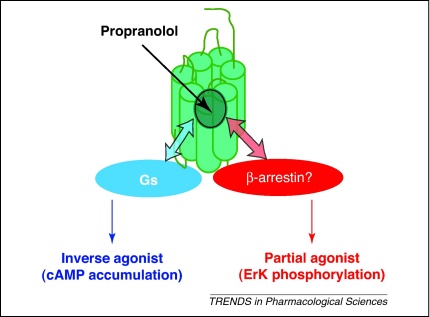
Biased signalling from the β_2_-adrenoceptor in response to propranolol in CHO-K1 cells [121]. The presence of different intracellular signalling proteins (e.g. Gαs or β-arrestin) bound to the β_2_-adrenoceptor provides an allosteric mechanism for reciprocal interaction between the signalling protein and the orthosteric ligand binding site. However, the extent to which a particular signalling pathway predominates will depend on both the affinity of ligands for particular receptor–effector complexes and the degree of compartmentalisation of these complexes within the cell.

When Sir James developed β-blockers, the idea of α- and β-adrenoceptor subtypes was quite radical. Three major groups of adrenoceptors are now identified that signal in characteristic patterns. α_1_-Adrenoceptors stimulate phospholipase C, whereas α_2_-adrenoceptors inhibit adenylyl cyclase and β-adrenoceptors activate it. However, in addition to the canonical signalling pathway, the nine adrenoceptor subtypes are now known to couple to other cell signalling mechanisms. It is also clear that linear efficacy (all agonists acting on the receptor in the same manner, with the only variables being affinity and efficacy) is an over-simplification [123]. Abundant evidence has now emerged indicating ligand-directed signalling bias, particularly among β-adrenoceptor antagonists, with the actions of propranolol at the β_2_-adrenoceptor being one of the best examples (Figure 1) [120,121].

Many β-blockers are actually weak partial agonists, and others are inverse agonists, at the β_2_-adrenoceptor in a variety of functional assays. Propranolol is an inverse agonist at the β_2_-adrenoceptor on the canonical cAMP pathway, but in the same cells it also produces a stimulatory Erk1/2 response (Figure 1) [120,121]. This is explained by the existence of several agonist–receptor conformations stabilised by an interaction with a specific signalling protein.

For example, a ligand binds to a transmembrane orthosteric site, which allosterically alters the receptor conformation that then determines the specificity of binding for the intracellular signalling protein (e.g. G protein or β-arrestin). This will occur in a ligand-dependent manner [124], and whether a ligand is considered an agonist, inverse agonist or neutral antagonist (rare) depends on the signalling pathways being examined. After cAMP, the most studied signalling pathways examined for β-adrenoceptor ligands are β-arrestin recruitment [125] and Erk1/2 signalling [120,121], but there is also evidence of tyrosine kinase receptor transactivation and p38 MAPK, PI3 kinase and NO activation, depending on the β-adrenoceptor subtype, level of receptor expression and cell type [123].

Several β-adrenoceptor ligands have complex efficacy profiles for cAMP generation and Erk1/2 activation at both β_1_- and β_2_-adrenoceptors, which is termed pluridimensional efficacy [110,120,121,126]. For mouse [127] and human [128] β_3_-adrenoceptors, SR59230A and L748337 are classical competitive antagonists for cAMP accumulation but agonists for Erk1/2 and p38 MAPK activation. This suggests that compounds selectively activate discrete pathways by interacting with particular receptor conformations. Although many β-blockers express their own spectrum of pharmacological properties, currently there is little to relate clinical efficacy with their ability to activate MAPK or other signalling pathways. Suggestions that the therapeutic benefit of carvedilol in heart failure patients relates to its capacity to activate Erk1/2 signalling by G-protein-independent mechanisms seems premature given that other β-blockers without these properties have similar clinical efficacy [125].

More extensive evaluations of β-blockers have examined their ability to stimulate cAMP production (using Exchange Protein Activated by Cyclic AMP (EPAC)-based biosensors) or Erk1/2 activation in cells expressing the β_2_-adrenoceptor [125]. A mutant β_2_-adrenoceptor with poor G protein coupling demonstrated that carvedilol remained a partial agonist for Erk1/2 activation, whereas propranolol produced no response [125]. The carvedilol Erk1/2 response was sensitive to siRNA depletion of arrestin-3, but insensitive to pertussis toxin (PTX). Thus, carvedilol (but not propranolol) causes receptor phosphorylation, recruitment of arrestin3-GFP, and receptor internalisation without changes in cAMP [125].

The β_1_-adrenoceptor also displays ligand-directed signalling bias. Treatment with isoprenaline caused both G_i_-dependent and G-protein-independent Erk1/2 activation [129]. Bucindolol was a partial agonist and propranolol was an inverse agonist for cAMP, but both caused Erk1/2 activation. The isoprenaline-induced Erk1/2 response was partially blocked by PTX or overexpression of beta Adrenergic Receptor Kinase-carboxy terminus (βARK-ct) (which sequesters Gβγ subunits), but responses to bucindolol and propranolol were unaffected [129]. Only receptors treated with isoprenaline produced a Bioluminescence Resonance Energy Transfer (BRET) signal, which suggests that isoprenaline, bucindolol and propranolol promote distinct conformations of the β_1_-adrenoceptor [129]. Although these findings suggest that Erk1/2 activation by β_1_-adrenoceptor ligands does not involve arrestins [129], it has been shown that carvedilol and alprenolol interact with the β_1_-adrenoceptor to promote arrestin-2/3 recruitment, transactivation of the EGF receptor and Erk1/2 activation [130], whereas propranolol does not stimulate arrestin recruitment (bucindolol was not tested) [129]. Thus, different drugs could have distinct modes of action, not only with respect to cAMP and Erk1/2 signalling, but also in terms of upstream signalling effectors. However, the carvedilol and alprenolol study used the mouse β_1_-adrenoceptor [130], whereas the propranolol and bucindolol study used the human β_1_-adrenoceptor [129], that have amino-acid differences that could differentially affect phosphorylation or arrestin recruitment.

At the mouse and human β_3_-adrenoceptors, drugs that act as antagonists of cAMP responses strongly activate Erk1/2 [127,128]. However, and in contrast to β_1_- and β_2_-adrenoceptors, the Erk1/2 responses at β_3_-adrenoceptors do not involve receptor phosphorylation, arrestin interaction or internalisation because the β_3_-adrenoceptor does not undergo any of these processes. In cells expressing modest levels of human β_3_-adrenoceptors, L748337 was a competitive antagonist for cAMP accumulation, but had high agonist potency and efficacy for Erk1/2 phosphorylation [128]. Zinterol, by contrast [131], had high efficacy for cAMP accumulation but lower efficacy than L748337 for both Erk1/2 and p38MAPK phosphorylation [128]. Efficacy reversal was also demonstrated for CL316243 and SR59230A at mouse β_3_-adrenoceptors [127]. For cAMP accumulation, CL316243 was a full agonist and SR59230A either a partial agonist or antagonist, depending on receptor expression levels. In identical cells but using the extracellular acidification rate as the functional readout, both CL316243 and SR59230A are full agonists at all levels of receptor expression. Analysis of responses using selective MAPK inhibitors and Western blotting showed that p38 MAPK and Erk1/2 signalling are involved and confirmed that SR59230A has much higher efficacy than CL316243 for MAPK signalling. Such examples of reversal of efficacy provide strong support for ligand-directed signalling bias.

At β_3_-adrenoceptors, MAPK responses induced by agonist ligands occur via different G proteins from those induced by antagonist ligands. Thus, Erk1/2 activation by L748337 is blocked by PTX, which indicates that G_i/o_ but not G_s_ is involved, whereas activation in response to L755507 is much less affected by PTX, suggestive of coupling predominantly to G_s_ [128]. This again highlights differences between the three human β-adrenoceptors because antagonist-stimulated Erk1/2 phosphorylation at the β_1_- and β_2_-adrenoceptors is not PTX-sensitive [125,129].

Important factors to consider in studying pathway-specific pharmacology include the level of receptor expression and the conditions under which each signalling pathway is measured. Signalling assays used to demonstrate ligand-directed signalling bias are often conducted under different conditions and rarely at equilibrium. cAMP accumulation assays are often conducted with phosphodiesterase inhibitors present. Erk1/2 assays involving Gβγ or receptor interaction with cSrc tend to peak at 5–8 min, whereas those involving β-arrestin interactions have longer time courses. Concentration–response relationships usually involve picking the response peak for a range of concentrations. Likewise, Ca^2+^ assays often follow a peak then plateau phase. By contrast, β-arrestin responses and reporter gene assays are followed over minutes/hours. The widely varying conditions under which assays are conducted could play a role in the effects observed. However, comparisons are generally made with a reference agonist, generally the physiological ligand.

## Quantitation of ligand-directed signalling bias

For ligand-directed signalling bias to be useful in drug development, methods for quantitation are required. These have recently become available and, together with the identification of drug profiles associated with therapeutic efficacy, provide a way forward for the development of new classes of selective drugs. Interestingly, the starting point for quantitation of bias is the Black–Leff operational model [2,132]. Bias must encompass both affinity (*K*_A_) and efficacy (τ) and the ratio τ/K_A_ is referred to as the transduction ratio [133]. For each bioassay, transduction ratios can be determined and compared to those obtained for a reference, usually an endogenous, agonist. By setting the τ/*K*_A_ value for the reference agonist to 1, comparisons can be made of the bias factors for other agonists across bioassay systems. This facilitates systematic comparison of ligands acting at target GPCR systems. One advantage of bias factor measurement is that rather than examining systems for extreme behaviour (e.g. β-adrenoceptor antagonists that are Erk1/2 activators), it is possible to examine many of compounds that display a range of different efficacies for different signalling pathways [134].

## Concluding remarks

The development of β-blockers by Sir James Black made a much greater contribution to pharmacology than originally imagined. They have proved much more useful than their original role of improving symptoms and outcome in ischaemic heart disease, a development that both interested and amused him in later years. Not only did he produce a new class of drugs, he also made a major contribution to analytical pharmacology by developing approaches that are now providing the foundations for the analysis of ligand-directed signalling bias. It is possible that the therapeutic applications of β-blockers in cardiac arrhythmias, hypertension and cardiac failure can be further enhanced by improvements in selectivity and exploitation of new properties. Novel applications for β-blockers in the treatment of cancer, osteoporosis, infectious disease and asthma will need further experimental support.

## Figures and Tables

**Table 1:** Current indications for β-blockers from the British National Formulary September 2010 (www.bnf.org)

Propranolol	Hypertension, ischaemic heart disease (IHD), arrhythmias, portal hypertension, anxiety, essential tremor, migraine, thyrotoxicosis
Acebutolol	Hypertension, IHD, arrhythmias
Atenolol	Hypertension, IHD, arrhythmias, migraine
Bisoprolol	Hypertension, IHD, heart failure
Carvedilol	Hypertension, IHD, heart failure
Celiprolol	Hypertension
Esmolol	Arrhythmias (short-term)
Labetolol	Hypertension
Metoprolol	Hypertension, IHD, arrhythmias, migraine
Nadolol	Hypertension, IHD, arrhythmias, migraine, thyrotoxicosis
Nebivolol	Hypertension, heart failure
Oxprenolol	Hypertension, IHD, arrhythmias, anxiety
Pindolol	Hypertension, IHD
Sotalol	Arrhythmias
Timolol	Glaucoma, hypertension, IHD, migraine
Betaxolol	Glaucoma
Carteolol	Glaucoma
Levobunolol	Glaucoma
Metipranolol	Glaucoma

## Footnotes

* β-Blocker is the commonly used term for β-adrenoceptor antagonist as defined by NC-IUPHAR. The term favoured by Sir James Black was β-receptor antagonist.

† Ligand-directed signalling bias is a process whereby different intracellular signalling cascades are differentially stimulated by different ligands via a single receptor subtype.
